# Benefit in liver transplantation: a survey among medical staff, patients, medical students and non-medical university staff and students

**DOI:** 10.1186/s12910-018-0248-7

**Published:** 2018-02-12

**Authors:** Christine Englschalk, Daniela Eser, Ralf J. Jox, Alexander Gerbes, Lorenz Frey, Derek A. Dubay, Martin Angele, Manfred Stangl, Bruno Meiser, Jens Werner, Markus Guba

**Affiliations:** 1 Department of General, Visceral, Vascular and Transplant Surgery, Klinikum der Universität München, Marchioninistrasse 15, 81377 München, Germany; 2 Department of Psychiatry and Psychotherapy, Klinikum der Universität München, Nußbaumstraße 7, 80336 München, Germany; 3 Institute of Ethics, History and Theory of Medicine, LMU Munich, Lessingstr. 2, 80336 München, Germany; 4 Department of Medicine II, Klinikum der Universität München, Marchioninistrasse 15, 81377 München, Germany; 5 Department of Anesthesiology, Klinikum der Universität München, Marchioninistrasse 15, 81377 München, Germany; 6 Transplant Center Munich, Klinikum der Universität München, Marchioninistrasse 15, 81377 München, Germany; 7 Department of Surgery, Division of Transplant Surgery, Medical University of South Carolina, 96 Jonathan Lucas Street, Charleston, SC 29425 USA

**Keywords:** Liver transplantation, Allocation, Urgency, Utility, Willingness to donate, Legal aspects, Quality of life, Ethics, Prospect of success, Benefit

## Abstract

**Background:**

The allocation of any scarce health care resource, especially a lifesaving resource, can create profound ethical and legal challenges. Liver transplant allocation currently is based upon urgency, a sickest-first approach, and does not utilize capacity to benefit. While urgency can be described reasonably well with the MELD system, benefit encompasses multiple dimensions of patients’ well-being. Currently, the balance between both principles is ill-defined.

**Methods:**

This survey with 502 participants examines how urgency and benefit are weighted by different stakeholders (medical staff, patients on the liver transplant list or already transplanted, medical students and non-medical university staff and students).

**Results:**

Liver transplant patients favored the sickest-first allocation, although all other groups tended to favor benefit. Criteria of a successful transplantation were a minimum survival of at least 1 year and recovery of functional status to being ambulatory and capable of all self-care (ECOG 2). An individual delisting decision was accepted when the 1-year survival probability would fall below 50%. Benefit was found to be a critical variable that may also trigger the willingness to donate organs.

**Conclusions:**

The strong interest of stakeholder for successful liver transplants is inadequately translated into current allocation rules.

## Background

Organ allocation often implies life and death decisions and thus has to be based on medically reasonable and ethically justified grounds. Liver allocation systems currently rely on algorithms focusing on urgency, a sickest-first approach. In an optimal case an urgency-driven allocation system would offer an organ at an optimal time point in the course of the disease and no waiting list deaths would occur. At present we are far from optimal.

In 2006 the Model of End-Stage Liver Disease (MELD) allocation system was adopted in Germany. The MELD score is an urgency-based quantifiable allocation system based only upon serum total bilirubin, serum creatinine and the international normalized ratio (INR) and correlates well with wait-list mortality [1–3]. After MELD introduction in Germany, the average MELD score for a regular liver allocation went up from 25 to meanwhile 34. Although higher MELD scores are associated with increased waitlist mortality, post-transplant survival is decreased with MELD scores > 30 [3, 4].

Overall post-transplant survival has thus decreased in Germany, triggering a discussion that capacity to benefit should be integrated into liver transplant allocation algorithms [5, 6]. At present this discussion is ongoing and implies not only medical considerations, but also ethical, legal and social aspects.

With this survey we wanted to describe how urgency and benefit are weighted by the stakeholders in order to enrich the discussion how these two criteria should be balanced in a liver allocation system.

## Methods

### Respondents

The survey was conducted on 4 separate groups: 1) Medical staff consisting of physicians and surgeons, nurses and medical assistants working to various degrees in transplantation medicine. Participants were approached during morning conferences and asked to anonymously fill out a paper-and-pencil questionnaire; 2) Patients with end-stage liver disease (ESLD) who either had received a liver transplant or were listed for a transplant. These patients were asked to fill out the questionnaire while waiting in the transplant outpatient clinic; 3) Medical students in their third to fifth year, who were approached at University after their lecture; and 4) Non-medical university staff and students, who were approached by a web-based survey tool.

The response rate of the paper-based survey was 70.4% (342 of 486 questionnaires completed and returned) which was used for groups 1–3. Information was missing in 17 questionnaires that prohibited categorization to one of the above mentioned groups and thus were excluded from analysis. For group 4, non-medical university staff and students were approached using an online survey tool (LimeSurvey) linked to the university information service and subscribed by 5489 users. The online questionnaire was answered by 115 respondents from group 4. There also were 28 additional respondents from group 1 and 34 from group 3 who completed the online survey. In total, there were 502 completed surveys available for analysis.

In group 1 (medical staff) 24 participants worked at the hospital and studied medicine, in group 2 (patients), 7 patients pointed out to have a medical background. Three patients waiting for a re-transplant were categorized in the pre-transplant group.

Data were collected from February to July 2015. A declaration of no-objection for the survey was granted by the ethics committee of the LMU Munich.

### Study design

In preparation of the survey, a pilot survey was performed with 3 persons of each group to ensure reading comprehensibility. The final product contained 7 quantitative survey questions regarding capacity to benefit in liver transplantation and additional 4 questions regarding the national liver allocation system in Germany (not included in this publication). Following the survey questions, basic demographic information was obtained from participants that may influence the attitude towards organ transplantation, including age, gender, the highest level of education, working in the field of transplantation medicine, medical profession (physician, medical assistant, nurse and medical student), patient status (post-transplant or listed for transplant), current state of health, smoking, body mass index (BMI), and religiosity [6–9].

### Analysis

Data were analyzed using SPSS Statistics 23 (IBM Corporation, Armonk, New York, USA). An ordinal logistic regression model (proportional odds model) was used to analyze the influence of personal factors on the balance of urgency and benefit. For analysis the five point scale was contracted. Due to missing answers, the sample size for the regression analysis decreased to *n* = 397. Variables with a significant difference of *p* < 0.25 were selected for the ordinal regression. *P*-values (Wald-test) of *p* < 0.05 were considered significant. The sample size was sufficient to perform the analysis on the above mentioned influencing factors [10].

## Results

**Fig. 1:**
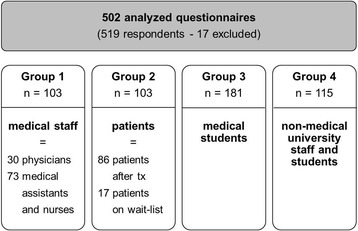
Classification of respondents

The study population including the subgroups is depicted in Fig. 1. Group 1 (medical staff) included more medical assistants and nurses but less physicians. In group 2 (ESLD patients) the majority of respondents had already undergone a liver transplant.

Demographics are shown in Table 1. Group 2 (ESLD patients) were older, had a lower performance status, lower education level, but they generally tended to be more religious as compared to the remaining groups 1, 3 and 4.

**Table 1:** Personal data, n (%)

	Medical staff	Patients	Medical students	Non-medical persons	Total of respondents
Mean age	31.8	55.2	23.1	30.3	32.0; Range: 19–80
Gender					
Male	40 (38.8)	69 (67.6)	68 (37.6)	34 (29.6)	211 (42.1)
Female	63 (61.2)	33 (32.4)	113 (62.4)	81 (70.4)	290 (57.9)
Current state of health					
ECOG 0	99 (97.1)	34 (34.3)	177 (98.3)	109 (94.8)	419 (84.5)
ECOG 1	3 (2.9)	39 (39.4)	2 (1.1)	6 (5.2)	50 (10.1)
ECOG 2	0 (0.0)	24 (24.2)	1 (0.6)	0 (0.0)	25 (5.0)
ECOG 3	0 (0.0)	2 (2.0)	0 (0.0)	0 (0.0)	2 (0.4)
ECOG 4	0 (0.0)	0 (0.0)	0 (0.0)	0 (0.0)	0 (0.0)
Smoking					
Yes	19 (18.4)	17 (16.5)	14 (7.7)	6 (5.2)	56 (11.2)
No	84 (81.6)	86 (83.5)	167 (92.3)	109 (94.8)	446 (88.8)
Body mass index					
< 18.5	1 (1.1)	3 (3.2)	7 (3.9)	10 (8.8)	21 (4.4)
18.5–24.9	70 (74.5)	41 (43.2)	155 (87.1)	75 (66.4)	341 (71.0)
25–29.9	16 (17.0)	32 (33.7)	16 (9.0)	21 (18.6)	85 (17.7)
>/= 30	7 (7.4)	19 (20.0)	0 (0.0)	7 (6.2)	33 (6.9)
Highest completed level of education					
Basic secondary (Hauptschule)	1 (1.0)	39 (39.8)	1 (0.6)	1 (0.9)	42 (8.5)
Advanced secondary (Realschule)	14 (13.9)	27 (27.6)	0 (0.0)	4 (3.5)	45 (9.1)
Final secondary (Hochschulreife)	32 (31.7)	9 (9.2)	159 (88.8)	52 (45.2)	252 (51.1)
College (Fachhochschule)	11 (10.9)	10 (10.2)	3 (1.7)	5 (4.3)	29 (5.9)
University (Universität)	43 (42.6)	13 (13.3)	16 (8.9)	53 (46.1)	125 (25.4)
Considering oneself a religious person					
Yes	54 (52.9)	72 (74.2)	91 (51.7)	58 (50.4)	275 (56.1)
No	48 (47.1)	25 (25.8)	85 (48.3)	57 (49.6)	215 (43.9)
Working in the field of transplantation medicine					
Yes	53 (51.5)	0 (0.0)	16 (8.8)	0 (0.0)	69 (13.9)
No	50 (48.5)	99 (100.0)	165 (91.2)	115 (100.0)	429 (86.1)
Willingness to donate organs					
Yes	84 (82.4)	92 (92.0)	149 (82.8)	85 (73.9)	410 (82.5)
No	14 (13.7)	4 (4.0)	13 (7.2)	11 (9.6)	42 (8.5)
I do not know	4 (3.9)	4 (4.0)	18 (10.0)	19 (16.5)	45 (9.1)

### Criteria for success: Gain in lifetime and gain in quality of life

*In your opinion, what is the criterion for success in liver transplantation? (Possible answers: 1) Gain in lifetime, 2) Gain in quality of life, or 3) Both lifetime and quality of life).*

The vast majority of respondents stated that both – gain in lifetime and gain in quality of life – are criteria for success (436/502 = 86.9%). Surprisingly, there was a considerable portion of 20.0% (23/115) of respondents in group 4 (non-medical persons) who emphasized only gain in quality of life.

### Gain in lifetime

*Let’s suppose that gain in lifetime is a criterion for success: How long at a minimum should a patient survive after transplantation in order to call it “successful”? (Possible answers: several hours, several days, 3 months, 1 year, 5 years, or 10 years).*

**Fig. 2:**
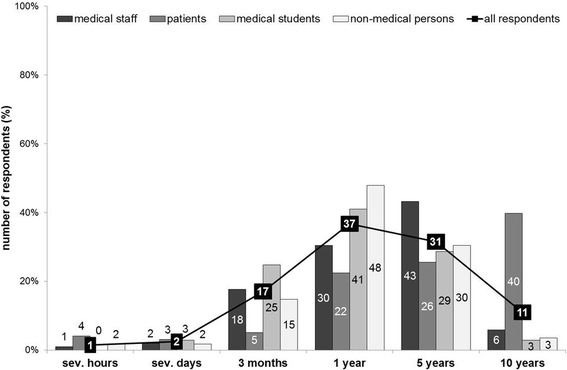
Gain in lifetime as criterion for “successful” liver transplantation

Collectively, a minimum survival of 1 year after a liver transplant was the most common choice to classify the transplant as successful (181/493 = 36.7%). Interestingly, medical staff and patients had higher expectations on the outcome of a transplant procedure;10 year survival was the most common interval chosen by patients (39/98 = 39.8%) and 5 year survival was the most common interval chosen by medical staff (44/102 = 43.1%).The answers are detailed in Fig. 2.

### Performance status

*Let’s suppose that gain in quality of life is a criterion for success: Which quality of life regarding independence and mobility would you expect at a minimum after transplantation in order to call it “successful”? (Possible answers based on the well-defined ECOG performance status from ECOG 0 (fully active, all performance without restriction) to ECOG 4 (completely disabled, totally confined to bed or chair)* [11]).

To call a liver transplant successful, most respondents expected a performance status of ECOG 2 (ambulatory and capable of all self-care, but unable to carry out any work activities − 204/495 = 41.2%), or ECOG 1 (restricted in physically strenuous activity but ambulatory and able to carry out work of a light or sedentary nature - 173/495 = 34.9%). Respondents generally did not accept a state of disability and poor self-care; only few respondents chose ECOG 3 (capable of only limited self-care and confined to bed or chair more than 50% of waking hours - 61/495 = 12.3%) or ECOG 4 (3/495 = 0.6%).

**Fig. 3:**
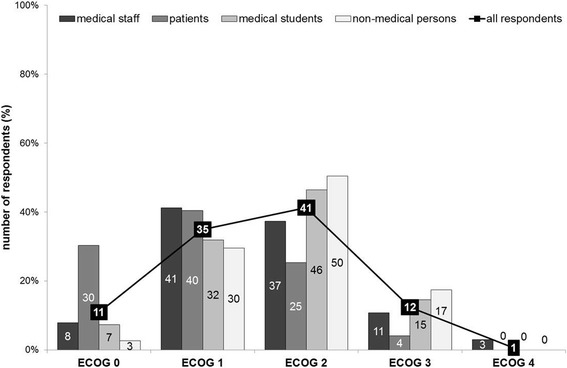
Gain in quality of life as criterion for “successful” liver transplantation

Patients had higher postoperative performance expectations as compared to all other groups; the most frequent answer was ECOG 1 (40/99 = 40.4%), followed by ECOG 0 (30/99 = 30.3%). All results are presented in Fig. 3.

### Acceptance of delisting

*As donor organs are limited, not all patients requiring a liver transplantation can be transplanted. At which risk of dying within 1 year after transplantation would you – as patient on the waiting list – accept delisting? (Possible answers ranged from a probability of death of 0% to 100%).*

**Fig. 4:**
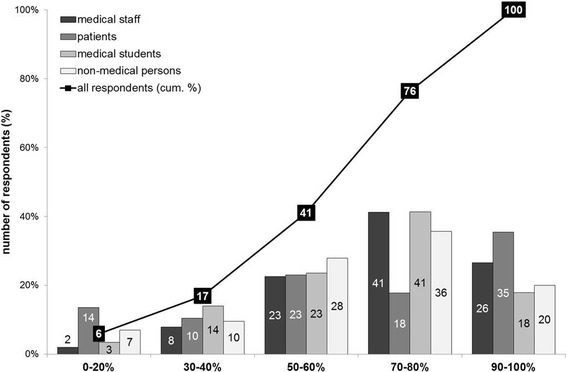
Acceptance of delisting with a probability of death of 0% to 100%

Delisting from the waiting list was generally accepted beginning with a post-transplant mortality risk of 50%. Numerically, the most common interval where respondents accepted delisting was a probability of death of 70–80% (174/492 = 35.4%). Again, patients answered differently as compared to the other groups. Most patients were willing to accept extremely poor outcomes; they did not accept being taken off the waiting list until a mortality risk of 90–100% (34/96 = 35.4%). See Fig. 4 for all results.

### Urgency versus prospect of success – a dilemma

*According to the German Transplant Law, prospect of success and urgency shall be considered in the organ allocation system (§ 12 (3) TPG). A balance between both interests is difficult to achieve in liver transplantation, due to the scarcity of donor organs: The most urgent patients are seriously ill when they receive the donor organ. Even if, in a first step, the transplantation has been successful, their survival afterwards is inferior. Patients with good prospect of success are, in most cases, not as sick yet. Therefore, they are not as urgent. They better cope with the transplantation and their survival is better. Should the most urgent patient - the one whose probability of death without transplantation is highest - get the next available organ in any case? Or should the patient with the best prospect of success - the one who statistically can survive for the longest period of time with the transplanted organ - get the next donor organ? Please weigh these two interests. (Urgency and prospect of success could be weighed across a five-point scale).*

**Fig. 5:**
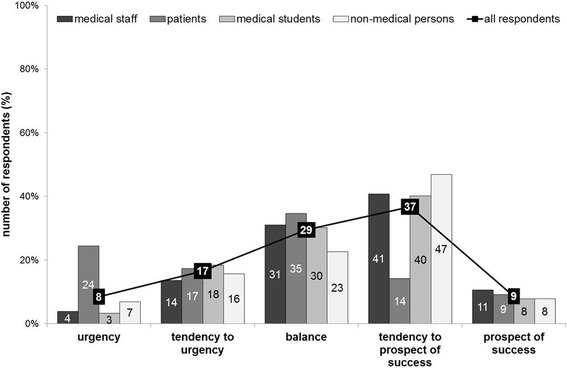
Urgency versus prospect of success – a dilemma

The most common response was a tendency to favor prospect of success (182/495 = 36.8%). Again, patients responded differently as compared to the other groups, voting for a balance of the two interests (34/98 = 34.7%) or urgency (24/98 = 24.5%) more often than the other groups. All results are displayed in Fig. 5.

### Influence of personal factors on the balance of urgency and prospect of success

We examined a possible influence of personal factors on the balance of urgency and prospect of success with an ordinal logistic regression analysis. Three variables had a statistically significant effect on the decision. First, persons working in the field of transplantation medicine were more likely to favor prospect of success compared to persons not working in the field (Wald = 13.474, *p* < 0.001, OR 0.24, 95% CI: 0.11 to 0.51). Second, smokers were more likely to consider urgency than nonsmokers (Wald = 5.879, *p* = 0.015, OR 2.23, 95% CI: 1.17 to 4.28) and patients were more likely to consider urgency than non-medical persons (Wald = 4.220, *p* = 0.040, OR 2.69, 95% CI: 1.05 to 6.91).

Other personal factors such as age, gender or education had no statistically significant effect on the balance between urgency and prospect of success in our survey.

### Influence of prospect of success on willingness to donate organs

First, participants were asked if they were currently willing to donate their organs after death. Patients were more willing to donate their organs after death than participants of all other groups. All results can be seen in Table 1.

*Does the prospect of success of the performed transplantations influence your decision whether or not to donate your organs after your death? If influence yes: In what way? (Possible answers: I would like to donate my organs only/rather if they are given to patients with a high prospect of success/urgency).*

**Fig. 6:**
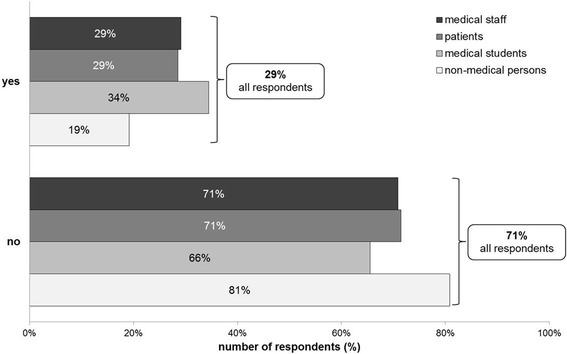
Influence of prospect of success on willingness to donate organs

**Fig. 7:**
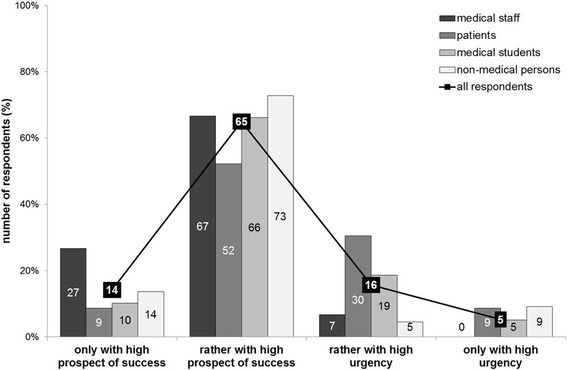
If influence yes: I would like to donate my organs only/rather if given to patients with high prospect of success/urgency

Most respondents denied that an influence of prospect of success would affect their willingness to donate organs (349/489 = 71.4%). Interestingly, 28.6% (140/489) of the respondents affirmed an influence of prospect of success would affect their willingness to donate. The latter were asked how prospect of success influenced their decision. The great majority rather wanted to donate their organs if they were given to patients with a high prospect of success (87/134 = 64.9%). See Figs. 6 and 7 for all results.

## Discussion

Benefit in liver transplantation is the focus of this survey. The goal was to investigate the role of capacity to benefit in the allocation system of liver grafts. It is challenging to allocate organs in times of organ shortage from a medical, ethical and legal standpoint. The problem of organ shortage is exacerbated in Germany by the fact that organ donation numbers are lower than in other countries [12]. Several allocation criteria are discussed and the question arises whether a satisfying balance can be found between them. Allocation of liver grafts competes with two principles: urgency and capacity to benefit. Urgency of need is also known as the “sickest-first” principle. In this case, the most urgent patient, the one whose probability of death without transplantation is highest, gets the next available organ. These patients are extremely ill when they receive the donor organ, which often negatively impacts post-transplant survival. In contrast, benefit focuses on patients who have the better post-transplant survival. In most cases, these patients are not as ill at the time of transplantation [5, 13, 14].

The question arises whether and to what extent benefit should be weighted in a liver allocation system. Adding benefit to the liver allocation system is currently limited by the lack of an accurate quantitative measure of the benefit criteria. Diverse definitions or target criteria of benefit exist [5, 15]. Benefit is best understood as an utilitarian concept as a criterion that favors the maximization of net benefit for the highest possible number of patients. This general rule, however, can be operationalized in different ways, for example, save the most lives of individual patients, save the most life years of the group of patients [5, 13, 16, 17] or maximize the survival benefit of them (difference of lifetime with vs. without a liver transplant) [18–23]. The German Transplant Law does not define benefit. Guidelines of the German Medical Association (GMA) specify the German Transplant Law. These guidelines define benefit as longer-term sufficient transplant function translating into a longer-term survival of the recipient with an improved quality of life [2].

We investigated the GMA definition of benefit, focusing on longer-term survival and improved quality of life. The vast majority of survey respondents chose gain in lifetime and gain in quality of life as the fundamental criteria of successful liver transplantation. The aim was to find out what longer-term survival and improved quality of life means for the different groups. This survey shows that affected patients, medical staff, medical students and non-medical university staff and students had a clear and relatively uniform idea about benefit in liver transplantation. The majority of respondents considered liver transplantation successful if there was a gain in lifetime of more than a year. Compared to non-medical persons, the medical staff and patients even had higher expectations with at least a five or 10 year increased survival, respectively.

An important dimension of quality of life is the ability to live a self-determined, independent life. We have based our question on the definition of the ECOG performance status [11] in order to facilitate a description of the abstract concept “quality of life”. Most respondents expected a performance status with at most ECOG 2. Being ambulatory and capable of all self-care was important to call a liver transplant successful. Interestingly, liver transplant patients had the highest postoperative performance status expectations.

The current MELD-based liver allocation system only adopts the urgency principle [1, 2]. This current practice of allocating livers was confirmed by previous studies on allocation criteria that found a preference for urgency-based allocation [6, 7, 14, 24, 25]. Some legal scholars suggest that urgency is the only category which should be used for allocation of life chances [16, 26]. Our results show that liver transplant patients favor the sickest-first allocation, although all other groups (medical staff, medical students, and non-medical participants) tended to favor benefit.

Previous studies on allocation criteria demonstrated that both – urgency and capacity to benefit – were important factors within an allocation system, some giving priority to benefit [9, 27–30]. According to the German Transplant Law, “Erfolgsaussicht” (literally translated as prospect of success) and urgency shall be considered (§ 12 (3) TPG). It has to be noted that prospect of success and capacity to benefit are mutually interchangeable. Benefit is more frequently used internationally. A committee of the German Medical Association determines specific allocation guidelines for each organ, specifying the German Transplant Law. In contrast to the current liver allocation system, the lung allocation scheme incorporates benefit in addition to urgency in Germany. The lung allocation system is based on the Lung Allocation Score (LAS), which weighs both aspects in the allocation algorithm. Benefit is integrated through taking into account the estimated survival probability and projected duration of 1-year survival with or without a lung transplantation [31, 32]. The US has recently adopted a utility-based allocation system for kidney transplantation as well based upon the Estimated Post-Transplant Survival (EPTS) scoring system. Therefore, benefit is incorporated through taking into account the estimated post-transplant survival [33]. Similar to the LAS and EPTS, most respondents in this study wanted both urgency and benefit criteria incorporated in the liver allocation system. In fact, several of the survey groups (medical staff, medical students and non-medical persons) favored benefit over urgency.

Benefit not only affects the allocation of liver grafts, but also influences (de) listing decisions. In times of organ shortage, not all patients requiring a transplant can be transplanted. We were interested whether respondents would accept a delisting decision when their mortality risk exceeds a defined threshold. Most respondents would only accept delisting with a 1-year mortality risk of 50%, significantly higher than the 25% 1-year post-liver transplant mortality observed in Germany [12]. One common survival “rule of thumb” is greater than 50% chance of 5-year survival post-transplant utilized in North America and Europe [23, 34–36], UK [30, 34] and Australia [14]. Currently, German delisting criteria for defining patients as “too ill” for transplant are not yet established. After Germany adopted the MELD score, there was a significant increase in the proportion of liver transplants performed on critically ill hospitalized patients. Early outcomes of liver transplantation declined precipitously [3, 12].

The final issue of this survey was the connection between benefit and the willingness to donate organs. Transplantation medicine has to rely on the general public’s willingness to donate organs. The general public needs to be represented in liver allocation decisions to achieve an accepted and supported allocation system [30, 37–39]. Previous studies showed a difference between the general public’s and the medical staff’s opinion [6, 37, 40] and only few studies approached the patient’s expectations and success criteria for liver transplantations [41, 42].

We asked whether benefit in liver transplantation would have an impact on the willingness to donate organs. Almost 30% of all respondents claimed that benefit would be a critical factor for their willingness to donate their organs.

This study shares limitations common to survey-based studies including representativeness and generalizability. This single center study consisted of university staff and students, which may not be an adequate sample of the general population. On average, respondents were younger and better educated than members of the general public. Also, the survey was limited to urgency of need and capacity to benefit. Other factors relevant for organ allocation such as time on the waiting list or reciprocity were not included. Finally, there were no tests of reliability or validity on the survey instrument.

## Conclusions

Despite these limitations, there are several conclusions that can be drawn from this survey: 1) the majority of respondents wanted benefit to be considered in the liver allocation algorithm; 2) liver transplant recipients were expected to recover to the state of being ambulatory and capable of all self-care (ECOG 2); 3) at least a 1-year survival was expected; 4) most respondents would accept a delisting decision when the probability of death would exceed 50% within the first year after transplantation; and 5) benefit may be a critical variable that triggers a person’s willingness to donate organs.

Although there is more research to be done to define and conceptualize the idea of benefit in liver transplantation, the present study may serve to stimulate the discussion about allocation criteria and the consideration of benefit both in medicine and society.

## Abbreviations

BMI: Body Mass Index
CI: Confidence interval
EPTS: Estimated post-transplant survival
ESLD: End-stage liver disease
GMA: German medical association
INR: International normalized ratio
LAS: Lung allocation score
MELD: Model of end-stage liver disease
OR: Odds ratio
